# Romosozumab Followed by Antiresorptive Treatment Increases the Probability of Achieving Bone Mineral Density Treatment Goals

**DOI:** 10.1002/jbm4.10546

**Published:** 2021-10-06

**Authors:** Felicia Cosman, Cesar Libanati, Cynthia Deignan, Zhigang Yu, Zhenxun Wang, Serge Ferrari, Jens‐Erik Beck Jensen, Pilar Peris, Francesco Bertoldo, Eric Lespessailles, Eric Hesse, Steven R Cummings

**Affiliations:** ^1^ Columbia University New York NY USA; ^2^ UCB Pharma Brussels Belgium; ^3^ Amgen Inc Thousand Oaks CA USA; ^4^ Geneva University Hospital Geneva Switzerland; ^5^ Copenhagen University Hospital Hvidovre Denmark; ^6^ Hospital Clinic, IDIBAPS University of Barcelona Barcelona Spain; ^7^ University of Verona Verona Italy; ^8^ Centre Hospitalier Régional d'Orléans University of Orléans Orleans France; ^9^ Institute of Molecular Musculoskeletal Research University Hospital, LudwigMaximilians‐University Munich Germany; ^10^ University of California San Francisco San Francisco Coordinating Center San Francisco CA USA

**Keywords:** ANABOLICS, ANTIRESORPTIVES, CLINICAL TRIALS, DXA, OSTEOPOROSIS

## Abstract

Increases in bone mineral density (BMD) with osteoporosis treatment are associated with reduced fracture risk. Increasing BMD is therefore a goal of osteoporosis therapy. Here, we compare the probability of achieving a *T*‐score of > −2.5 over 3 years at the total hip (TH) or lumbar spine (LS) in women with osteoporosis, ≥55 years of age, after the following treatment sequences: 1 year romosozumab followed by 2 years denosumab (FRAME and FRAME extension trials), 1 year romosozumab followed by 2 years alendronate, or alendronate‐only for 3 years (ARCH trial). Probabilities of attaining the BMD target within 1 year of treatment were also determined. At both skeletal sites, in women with a baseline *T*score ≥ −2.7, there was >50% probability of achieving the BMD target with any 3‐year regimen. The probability of achieving the target BMD in those with a baseline TH *T*score equal to −3.0 was 61% with romosozumab/denosumab, 38% with romosozumab/alendronate, and 9% with alendronate. In those with a baseline LS *T*score equal to −3.0, the probability of achieving a *T*‐score > −2.5 was 93% with romosozumab/denosumab, 81% with romosozumab/alendronate, and 55% with alendronate. With 1 year of treatment, in patients with a baseline TH *T*‐score equal to −2.7, the probability of reaching the target *T*score with romosozumab was 71% to 78% and 38% with alendronate. For patients with an initial LS *T*‐score equal to −3.0, the probability of achieving the target *T*‐score over 1 year was 85% to 86% with romosozumab and 25% for alendronate. Our findings suggest baseline BMD and the probability of achieving BMD *T*‐score goals are factors to consider when selecting initial treatment for patients with osteoporosis. As baseline *T*‐score falls below −2.7 (TH) and −3.0 (LS), alendronate has <50% likelihood of achieving a BMD goal above osteoporosis range, whereas these probabilities remain relatively high for regimens beginning with romosozumab. © 2021 The Authors. *JBMR Plus* published by Wiley Periodicals LLC on behalf of American Society for Bone and Mineral Research.

## Introduction

Current guidelines for the treatment of postmenopausal women with osteoporosis in the United Kingdom, United States, and Europe are not fully aligned.^(^ ^1^, ^2^, ^3^ ^)^ Traditionally, first‐line treatment has consisted of an antiresorptive (eg, alendronate, denosumab), but recent studies have highlighted the need to begin with anabolic therapy to obtain optimal treatment outcomes in individual patients.^(^ ^4^, ^5^, ^6^, ^7^, ^8^, ^9^, ^10^, ^11^ ^)^ Fracture risk has emerged as a key variable to help select the initial therapy.^(^ ^1^, ^3^ ^)^ In patients at very high risk of fragility fracture, including those with a recent fracture, available data and emerging guidelines are increasingly supportive of initial treatment with an anabolic agent.^(^ ^2^, ^3^, ^12^, ^13^ ^)^ Currently available anabolic treatment options include abaloparatide or teriparatide, administered for up to 2 years, and romosozumab, administered for 1 year. These treatments are usually followed by an antiresorptive treatment using bisphosphonates or denosumab.^(^ ^3^ ^)^

A recent meta‐regression analysis including 38 randomized controlled trials (RCTs) linked the magnitude of treatment‐related changes in bone mineral density (BMD) with anti‐fracture efficacy, particularly for vertebral and hip fractures.^(^ ^14^ ^)^ Although low BMD is certainly not the only factor contributing to fracture risk, studies have confirmed the importance of the relationship between BMD level attained after or during therapeutic intervention and fracture risk reduction.^(^ ^5^, ^15^, ^16^, ^17^, ^18^ ^)^ Overall, the findings suggest that total hip BMD has the strongest association with subsequent fracture risk and may be a useful clinical target for a goaldirected treatment approach for patients with osteoporosis.^(^ ^5^, ^15^, ^18^, ^19^, ^20^ ^)^

A total hip *T*‐score above −2.5 has been suggested as a minimum treatment goal, but higher total hip BMD targets may be more appropriate for women with additional independent fracture risk factors such as advanced age, underlying disease, or frequent falls.^(^ ^15^, ^16^, ^19^, ^21^ ^)^ As such, it is critical that treatment goals are tailored to the patient, with treatment decisions individualized accordingly.^(^ ^19^ ^)^ Goal‐directed therapy may be a useful concept to communicate the need for aggressive, early therapy for some patients with very low BMD or very high imminent fracture risk, and for critically evaluating initial treatment decisions. Finally, sharing a specific therapeutic target with the patient could be useful for optimizing adherence to therapy.

The bone‐forming agent romosozumab, approved for treating postmenopausal osteoporosis by FDA, EMA, PMDA, and other health authorities, is a humanized monoclonal antibody to the osteocyte protein sclerostin. By inhibiting sclerostin activity, romosozumab exerts a dual effect to stimulate modeling‐based bone formation and inhibit bone resorption.^(^ ^8^, ^22^, ^23^ ^)^ Because of its dual effect, romosozumab produces substantial gains in BMD over short periods of time, making it favorably indicated in patients at high imminent risk of fracture.^(^ ^22^, ^23^ ^)^ The most recent guidelines from the American Association of Clinical Endocrinologists (AACE) now include romosozumab treatment within their recommended first‐line treatment options for postmenopausal women at very high risk of fracture (eg, with prior fracture or very low BMD *T*score [< −3.0]).^(^ ^3^ ^)^

Since BMD on therapy serves as an indicator of treatment success, one of the factors that should be considered to determine choice of treatment is the probability of achieving specific BMD treatment targets. The primary objective of these post hoc analyses was to compare the probabilities of achieving a *T*‐score > −2.5 at the total hip or lumbar spine over 3 years with alendronate treatment alone versus treatment sequences of 12 months of romosozumab followed by 2 years of alendronate, or 12 months of romosozumab followed by 2 years of denosumab. An additional objective was to compare the probabilities of achieving non‐osteoporotic BMD *T*‐scores over 1 year of treatment with romosozumab compared with alendronate.

## Methods

### Study design

**Fig. 1:**
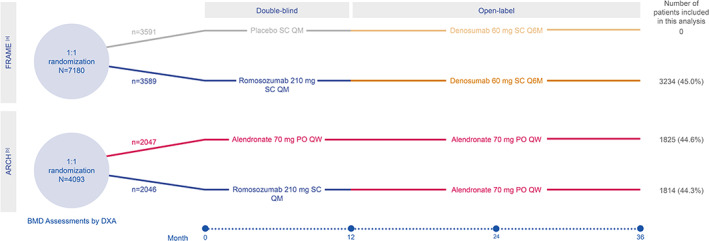
Study design. (*A*) Data from placebo arm of FRAME study not included in analyses. FRAME study extension comprised months 24 to 36. Enrolled participants included postmenopausal women aged 55 to 90 years with a total hip (TH) or femoral neck (FN) bone mineral density (BMD) *T*‐score ≤ −2.5 to −3.5. Key exclusion criteria included BMD *T*‐score ≤ −3.5 at the TH or FN, prior hip fracture (fx) or any severe or >2 moderate vertebral fracture (VFx). (*B*) Eligible participants included postmenopausal women aged 55 to 90 years with either (1) TH or FN BMD *T*‐score ≤ −2.5 and either ≥1 moderate/severe VFx or ≥2 mild VFx, or (2) TH or FN BMD *T*score ≤ −2.0 and ≥2 moderate/severe VFx or proximal femur fx within 3 to 24 months before randomization. Key exclusion criteria included contraindications to alendronate. For both FRAME and ARCH studies, additional key exclusion criteria included history of metabolic bone disease or use of agents affecting bone metabolism. DXA = dual‐energy X‐ray absorptiometry; PO = orally; QM = monthly; Q6M = every 6 months; QW = weekly; SC = subcutaneously.

These post hoc analyses were based on the FRAME (NCT01575834) and ARCH (NCT01631214) phase 3, multicenter, international, randomized, double‐blind trials in postmenopausal women with osteoporosis (Fig. 1).^(^ ^8^, ^22^ ^)^ {FIG1}Briefly, women in the FRAME trial received either romosozumab or placebo for 1 year, each followed by open‐label denosumab for 2 years (1 year in FRAME and a second year in the study extension).^(^ ^22^ ^)^ In ARCH, women received either romosozumab or alendronate for 1 year, followed by 2 years of open‐label alendronate in both groups; further study details have been previously described.^(^ ^8^ ^)^

Inclusion criteria for the two studies differed, with the criteria for ARCH requiring prior vertebral or proximal femur fracture, whereas those without history of fracture were eligible for participation in FRAME.^(^ ^8^, ^22^ ^)^ FRAME did not include women with a baseline total hip or femoral neck *T*‐score ≤ −3.5, whereas ARCH had no lower limit. Both studies required women to have a *T*‐score at the total hip or femoral neck below −2.5.

### Assessment and procedures

In the FRAME and ARCH studies, BMD at the proximal femur and lumbar spine were evaluated by dual‐energy X‐ray absorptiometry (DXA) at baseline and every 12 months thereafter using Hologic (Marlborough, MA, USA) or GE/Lunar (Madison, WI, USA) densitometers. Scans were processed and analyzed blinded to treatment at a central imaging vendor (Bioclinica, Princeton, NJ, USA) as previously described^(^ ^22^ ^)^; assessors remained blinded to treatment in both studies.

### Statistical analysis

All patients from the ARCH trial and women randomized to romosozumab from the FRAME trial, with total hip BMD values at baseline and at least one evaluable total hip BMD value at month 12 or 36, or with lumbar spine BMD values at baseline and at least one evaluable lumbar spine BMD value at month 12 or 36, were included in these post hoc analyses.

The probability of achieving a total hip or lumbar spine *T*‐score > −2.5 with the three treatment sequences at month 36 was estimated based on a logistic regression model, adjusted for baseline total hip or lumbar spine *T*‐score within each treatment group, respectively. Similar analyses were performed for probability estimates over 1 year with either romosozumab treatment (from either ARCH or FRAME) or alendronate treatment (from ARCH). In addition, the probability of achieving a total hip or lumbar spine *T*‐score > −2 with the three regimens at 1 and 3 years was analyzed because patients with high risk of fracture or other risk factors may warrant a higher BMD target to achieve an acceptably reduced risk of fracture. To deal with missing data, only patients with all the required variables available were included in each analysis (pairwise deletion). The baseline threshold *T*‐scores equal to −2.7, −3.0, and −3.5 were not prespecified. At a certain point for both total hip and lumbar spine, the probability of achieving a *T*score > −2.5 was below 50% when treated with alendronate alone. As such, these example baseline *T*‐score thresholds were selected logically to demonstrate a range of probabilities of achieving a *T*‐score > −2.5.

## Results

### Patient disposition

These post hoc analyses included 3234 women from the 3589 participants randomized to romosozumab/denosumab in the FRAME study, 1814 of the 2046 women randomized to romosozumab/alendronate in the ARCH trial, and 1825 of the 2047 women randomized to alendronate alone in the ARCH trial (Fig. 1). Consistent with the inclusion criteria for the FRAME and ARCH studies, baseline characteristics varied slightly between treatment groups, including slightly higher mean *T*‐scores for both total hip and lumbar spine among those enrolled in FRAME (romosozumab/denosumab: −2.5 and −2.7, respectively) compared with ARCH (romosozumab/alendronate: −2.8 and −2.9, respectively; alendronate only: −2.8 and −3.0, respectively) (Table 1). {TBL 1} In addition, the proportion of women who had baseline *T*scores ≤ −2.5 in the total hip were 53% in FRAME and 66% and 68% for those initially treated with romosozumab or alendronate in ARCH, respectively. The proportion of women with baseline lumbar spine *T*‐scores ≤ −3.0 were 42% in FRAME and 49% and 50% for those initially treated with romosozumab or alendronate in ARCH, respectively. Mean age was a few years older in ARCH versus FRAME (Table 1).

**Table 1:** Baseline Characteristics and Demographic Data

Baseline characteristics	FRAME	ARCH	ARCH
	Romo/Dmab	Romo/ALN	ALN
	(*N* = 3234)	(*N* = 1814)	(*N* = 1825)
Age (years), mean (SD)	70.7 (7.0)	74.2 (7.5)	74.0 (7.4)
BMD *T*‐score, mean (SD)			
Total hip^a^	−2.48 (0.47)	−2.77 (0.67)	−2.79 (0.65)
Lumbar spine^b^	−2.73 (1.04)	−2.94 (1.23)	−2.98 (1.22)
BMD *T*‐score, n (%)			
Total hip			
≤ −3.0	406 (12.6)	647 (35.7)	633 (34.7)
> −3.0 and ≤ −2.5	1311 (40.5)	556 (30.7)	597 (32.7)
> −2.5	1517 (46.9)	611 (33.7)	595 (32.6)
Lumbar spine			
≤ −3.0	1358 (42.0)	892 (49.2)	917 (50.2)
> −3.0 and ≤ −2.5	627 (19.4)	279 (15.4)	274 (15.0)
> −2.5	1173 (36.3)	573 (31.6)	555 (30.4)
Missing	76 (2.4)	70 (3.9)	79 (4.3)

Romo = romosozumab; Dmab = denosumab; ALN = alendronate; SD = standard deviation; BMD = bone mineral density.

Data from placebo arm of FRAME study not included in these analyses. *N* = Number of patients with total hip BMD values at baseline and at least one evaluable total hip BMD value at month 12 or month 36, or with lumbar spine BMD values at baseline and at least one evaluable lumbar spine BMD value at month 12 or month 36.

^a^ In patients with total hip BMD values at baseline and at least one evaluable total hip BMD value at month 12 or month 36, Romo/Dmab *n* = 3234, Romo/ALN *n* = 1814, ALN *n* = 1825.

^b^ In patients with lumbar spine BMD values at baseline and at least one evaluable lumbar spine BMD value at month 12 or month 36, Romo/Dmab *n* = 3158, Romo/ALN *n* = 1744, ALN *n* = 1746.

### Probabilities of achieving non‐osteoporotic BMD levels

#### Three years of treatment

After 3 years of treatment, during which those patients initially randomized to romosozumab had switched to either denosumab or alendronate, the probability of reaching a total hip or lumbar spine *T*‐score above −2.5 was dependent on treatment sequence.

**Fig. 2:**
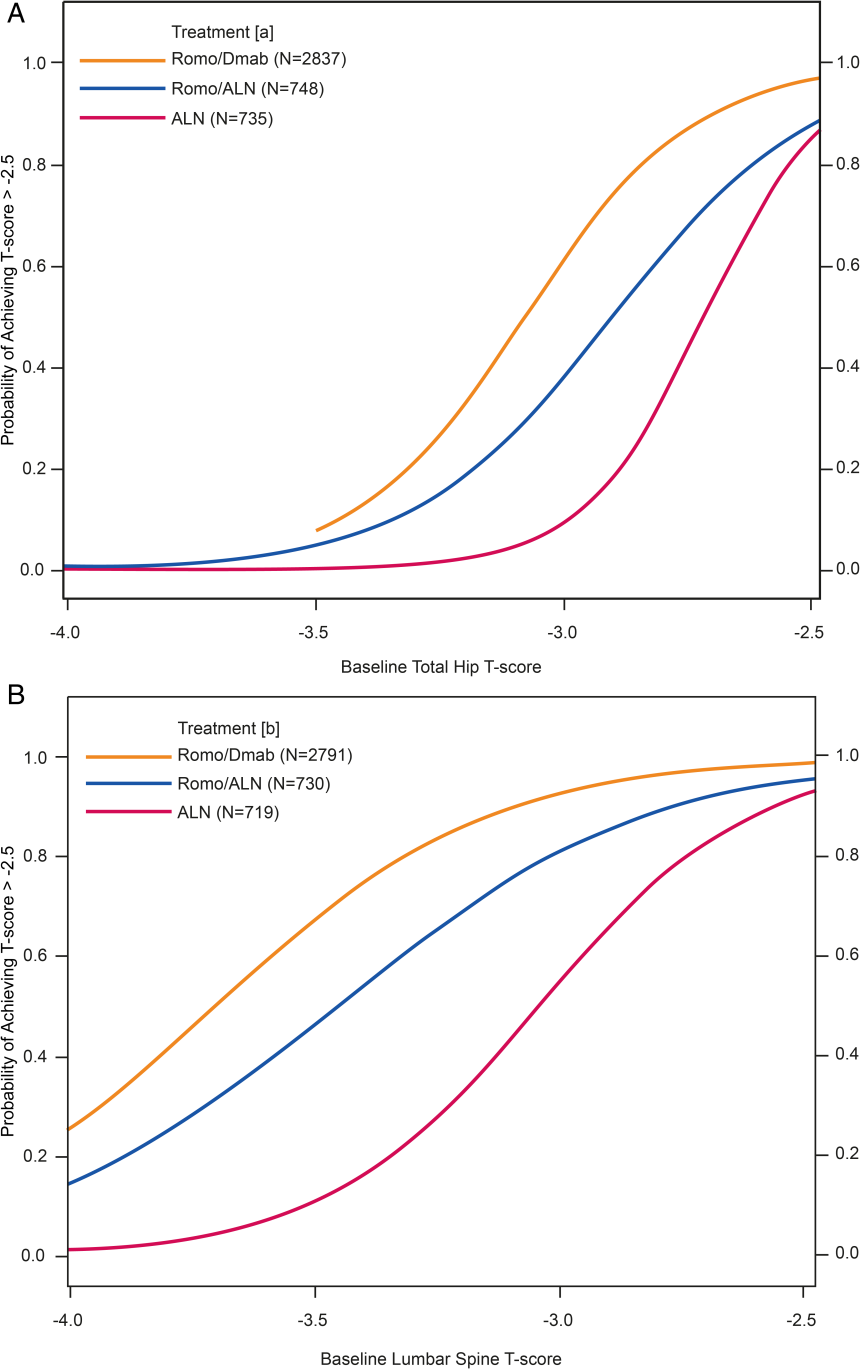
Probabilities of achieving a *T*‐score > −2.5 in the total hip (*A*) and lumbar spine (*B*) after 3 years of treatment with Romo/Dmab, Romo/ALN, or ALN only. (*A*) Number of patients with evaluable total hip bone mineral density (BMD) value at baseline and month 36. (*B*) Number of patients with evaluable lumbar spine BMD value at baseline and month 36. ALN = alendronate; Dmab = denosumab; Romo = romosozumab.

For patients with baseline *T*‐scores equal to or higher than −2.7 at the total hip, more than 50% of patients achieved a total hip *T*‐score > −2.5 within 3 years, regardless of treatment (Fig. 2*A*). {FIG2} For patients with baseline total hip *T*‐scores equal to −3.0, the probability of achieving a total hip *T*‐score above −2.5 was 61% with romosozumab/denosumab, 38% with romosozumab/alendronate, and 9% with alendronate alone. Patients with a baseline total hip *T*‐score equal to −3.5 had a low probability of reaching a total hip *T*score above −2.5 at 3 years with any of the treatment sequences (<10%; Table 2). {TBL 2}

**Table 2:** Probabilities of Achieving a *T*‐Score > −2.5 in the Total Hip and Lumbar Spine at Years 3 and 1 in Patients With Baseline *T*‐Scores Equal to −2.7, −3.0, and −3.5

Treatment	Baseline *T*‐score	Site of BMD measurement at year 3	Probability of *T*‐score > −2.5 at 3 years, % (95% CI)	Site of BMD measurement at year 1	Probability of *T*‐score > −2.5 at 1 year, % (95% CI)
ALN (*N* = 1825)	−2.7	Total hip (*n* = 735)^a^	53.3 (46.8, 59.7)	Total hip (*n* = 1778)^c^	38.4 (34.1, 42.8)
	−3.0		9.3 (6.2, 13.8)		3.5 (2.4, 5.1)
	−3.5		0.2 (0.1, 0.5)		0.0 (0.0, 0.1)
	−2.7	Lumbar spine (*n* = 719)^b^	82.6 (76.2, 87.5)	Lumbar spine (*n* = 1714)^d^	63.9 (58.8, 68.7)
	−3.0		54.8 (47.6, 61.7)		25.1 (21.0, 29.6)
	−3.5		11.0 (7.5, 15.9)		2.0 (1.3, 3.3)
Romo/ALN (*N* = 1814)	−2.7	Total hip (*n* = 748)^a^	72.8 (67.7, 77.4)	Total hip (*n* = 1773)^c^	70.7 (66.8, 74.4)
	−3.0		38.0 (32.6, 43.8)		21.5 (18.0, 25.5)
	−3.5		5.0 (3.1, 8.0)		0.7 (0.4, 1.2)
	−2.7	Lumbar spine (*n* = 730)^b^	91.5 (87.8, 94.2)	Lumbar spine (*n* = 1715)^d^	95.3 (93.5, 96.7)
	−3.0		80.6 (75.5, 84.9)		85.0 (81.6, 87.8)
	−3.5		45.9 (40.0, 51.8)		39.9 (35.5, 44.4)
Romo/Dmab (*N* = 3234)	−2.7	Total hip (*n* = 2837)^a^	90.0 (88.3, 91.5)	Total hip (*n* = 3186)^c^	77.6 (75.2, 79.8)
	−3.0		61.0 (57.5, 64.5)		23.8 (20.6, 27.3)
	−3.5		7.8 (5.6, 10.9)		0.6 (0.4, 0.9)
	−2.7	Lumbar spine (*n* = 2791)^b^	97.3 (96.4, 98.0)	Lumbar spine (*n* = 3140)^d^	96.5 (95.5, 97.4)
	−3.0		92.5 (90.9, 93.8)		85.8 (83.5, 87.9)
	−3.5		67.2 (64.2, 70.1)		32.3 (28.9, 35.8)

BMD = bone mineral density; CI = confidence interval; ALN = alendronate; Romo = romosozumab; Dmab = denosumab.

*N* = Number of patients with total hip BMD values at baseline and at least one evaluable total hip BMD value at month 12 or month 36, or with lumbar spine BMD values at baseline and at least one evaluable lumbar spine BMD value at month 12 or month 36.

^a^ Number of patients with evaluable total hip BMD value at baseline and month 36.

^b^ Number of patients with evaluable lumbar spine BMD value at baseline and month 36.

^c^ Number of patients with evaluable total hip BMD value at baseline and month 12.

^d^ Number of patients with evaluable lumbar spine BMD value at baseline and month 12.

In patients with an initial lumbar *T*‐score ≤ −2.7, there was a high probability of achieving a lumbar spine *T*‐score above −2.5 at 3 years with any of the treatments (97% with romosozumab/denosumab in FRAME, 92% with romosozumab/alendronate in ARCH, and 83% with alendronate alone) (Fig. 2*B*; Table 2). Among patients who started with a lumbar spine *T*‐score equal to −3.0, the probability of achieving a lumbar spine *T*score above −2.5 within 3 years was 93% with the romosozumab/denosumab sequence compared with 81% with the romosozumab/alendronate sequence and 55% for alendronate alone. For those with a lumbar spine *T*‐score equal to −3.5 at baseline, the probability of attaining a lumbar spine *T*score above −2.5 at 3 years was 67% with romosozumab/denosumab compared with 46% with romosozumab/alendronate and 11% with alendronate alone (Table 2).

#### One year of treatment

**Fig. 3:**
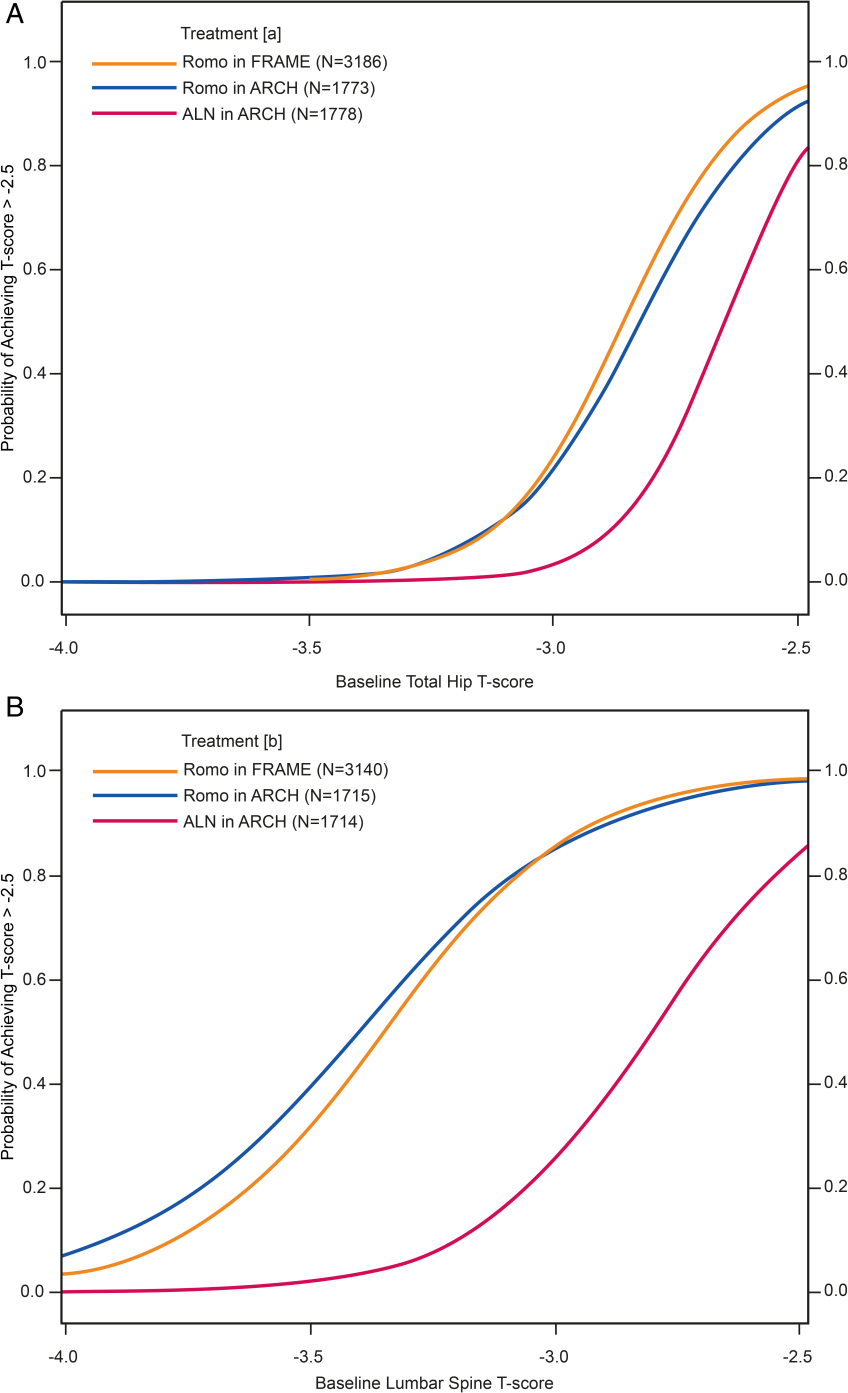
Probabilities of achieving a *T*‐score > −2.5 in the total hip (*A*) and lumbar spine (*B*) after 1 year of treatment with romosozumab or alendronate. (*A*) Number of patients with evaluable total hip bone mineral density (BMD) value at baseline and month 12. (*B*) Number of patients with evaluable lumbar spine BMD value at baseline and month 12. ALN = alendronate; Dmab = denosumab; Romo = romosozumab.

The probability of achieving a *T*‐score above −2.5 at total hip (Fig. 3*A*) {FIG3} or lumbar spine (Fig. 3*B*) was greater after 1 year of romosozumab treatment than 1 year of alendronate treatment.

In patients with a baseline total hip *T*‐score equal to −2.7, the probability of reaching a total hip *T*‐score > −2.5 was 78% and 71% with romosozumab (FRAME and ARCH, respectively) and 38% with alendronate. For patients with initial total hip *T*score equal to −3.0 or below, the probability of achieving a total hip *T*‐score above −2.5 after 1 year of either treatment was low (≤25%; Table 2).

In patients with a baseline lumbar spine *T*‐score equal to −2.7, the probabilities of attaining a lumbar spine *T*‐score above −2.5 were 97% and 95% for romosozumab treatment (in FRAME and ARCH, respectively) and 64% for alendronate. For patients with an initial lumbar spine *T*‐score equal to −3.0, the probabilities of achieving a lumbar spine *T*score > −2.5 were 86% and 85% for those treated with romosozumab in FRAME and ARCH, respectively, but only 25% for alendronate‐treated patients. In patients who began with a lumbar spine *T*‐score equal to −3.5, the probabilities of achieving a lumbar spine *T*‐score > −2.5 at 1 year were 32% and 40% for romosozumab‐treated patients (FRAME and ARCH, respectively) and 2% for alendronate‐treated patients.

### Probabilities of achieving BMD *T*‐score of ≥ −2.0 after 3 years

For patients with a baseline total hip *T*score equal to −2.7 treated initially with romosozumab, there was a 28% and 12% probability of achieving a *T*‐score ≥ −2.0 (followed by denosumab and alendronate, respectively) compared with 2% for alendronate‐only treatment. Patients with baseline total hip *T*‐scores below −2.7 had a low probability of achieving a total hip *T*‐score ≥ −2.0, regardless of treatment (<10%; Supplemental Table S1).

For patients with initial lumbar spine *T*‐scores equal to −2.7, the probability of achieving a lumbar spine *T*‐score ≥ −2.0 was 89% with romosozumab/denosumab, 68% with romosozumab/alendronate, and 39% with alendronate alone (Supplemental Table S1). The probability of achieving a lumbar spine *T*‐score ≥ −2.0 with initial romosozumab treatment for patients with baseline lumbar spine *T*‐scores equal to −3.0 was 71% and 47% (FRAME and ARCH, respectively) and 15% with alendronate alone (Supplemental Table S1). For patients with a baseline *T*‐score equal to −3.5, the probability of achieving a *T*score ≥ −2.0 was low across all treatments (≤25%).

## Discussion

In these analyses, we show that women with a baseline *T*‐score as low as −2.7 in the total hip or −3.0 in the lumbar spine have more than a 50% chance of achieving a *T*score above −2.5 with all three treatment regimens over 3 years. However, as baseline *T*‐scores fall below −2.7 to −3.0 in the total hip and below −3.0 to −3.5 in the lumbar spine, the probability of achieving a non‐osteoporotic BMD *T*‐score within 3 years of alendronate alone falls to about 10% at both sites; in contrast, the probabilities of achieving *T*‐scores above osteoporotic range remain high if the initial treatment is romosozumab.

With just 1 year of treatment in patients with baseline *T*‐scores of −2.7 or below in the total hip and −3.0 or below in the lumbar spine, the probability of attaining a nonosteoporotic BMD *T*score was low with alendronate alone. In contrast, with 1 year of romosozumab treatment, the probabilities of attaining non‐osteoporotic *T*scores were more than 70% for the total hip and about 85% for the lumbar spine. Whenever possible, women who have a baseline *T*‐score below −2.7 at the total hip or below −3.0 at the lumbar spine should be considered for romosozumab therapy first in order to provide a high likelihood of attaining BMD levels above the osteoporotic range within 1 to 3 years.

We investigated the probability of achieving a target *T*‐score > −2.5 because achieving this is a logical first step for patients who present with a BMD in the osteoporotic range. However, many fractures occur in women with *T*‐scores above −2.5,^(^ ^3^ ^)^ and for these and other individuals, attaining a *T*‐score of −2.0 or higher might be appropriate^(^ ^24^, ^25^ ^)^; we therefore also investigated the probability of achieving this BMD target. Although there was a lower probability of achieving this more stringent goal across all treatments, those treated initially with romosozumab had a much higher probability at both skeletal sites than alendronate alone. The goal of therapy should be amenable to personalization based on the presence of other independent risk factors for fracture. Having a goal can help close the recognized gap in long‐term adherence to treatment, supporting lifelong management of osteoporosis.

In patients who have a very high risk of fracture in the next 1 or 2 years, it is important to consider selection of a pharmacologic treatment that improves BMD and thereby reduces fracture risk as quickly as possible. In the FRAME trial, fewer fractures were observed in the second year, while all women were on denosumab, among patients who initially received romosozumab.^(^ ^5^ ^)^ Similarly, reduced fracture incidence was found in women originally randomized to romosozumab versus alendronate in the ARCH study during years 2 and 3, while all women were on alendronate. These findings suggest that the rebuilding of bone with romosozumab can lead to a persistent benefit after transition to an antiresorptive therapy. One year of treatment with romosozumab resulted in higher BMD compared with alendronate in the ARCH study, and higher BMD, particularly in the total hip, was associated with lower subsequent risk of both nonvertebral and vertebral fracture.^(^ ^25^ ^)^ In women who begin with a total hip *T*‐score of −2.7, 1 year of treatment with alendronate is unlikely to achieve the target BMD level, but the probability with 1 year of romosozumab exceeds 70%.

The results presented here also demonstrate that if a patient has a very low *T*score to begin with, particularly at the total hip (*T*‐score below −3.5), it may be difficult to achieve non‐osteoporotic BMD goals within 3 years, regardless of treatment. Still, a treatment strategy utilizing romosozumab initially followed by denosumab has the greatest probability of improving BMD to treatment targets within 3 years. Future research could illuminate whether multiple courses of romosozumab or other sequential, or even combination, treatments may provide further improvements.

### Study strengths and limitations

The large number of patients included in the FRAME and ARCH studies allowed a large sample size to be used in these post hoc analyses, increasing the robustness of the findings. Limitations included the different study designs of FRAME and ARCH. As the study design and eligibility criteria are different across the FRAME and ARCH studies, post hoc comparisons made across trials are indirect and descriptive. Additionally, in the present study, we did not analyze the relationship between the BMD achieved and the decrease in the risk of subsequent fracture, although this has been evaluated previously in ARCH and other studies.^(^ ^8^, ^16^, ^26^, ^27^, ^28^ ^)^ A further limitation of this study is that the analyses limit the achievement of improved BMD *T*‐scores to > −2.5 or ≥ −2.0. Achieving these BMD targets may not be adequate for patients who may have high fracture risk primarily from non‐BMD factors. Finally, it should be noted that here we assessed which treatment sequence was associated with the greatest chance of achieving a specific *T*‐score. Of course, the choice of therapeutic agent for each individual patient should take into consideration factors other than just BMD.

In patients with osteoporosis, treatment with romosozumab followed by an antiresorptive agent provided the greatest probability of achieving a target *T*‐score above osteoporosis range at the total hip and lumbar spine within 3 years, with the romosozumab/denosumab sequence yielding the greatest probability of achieving this goal in patients with the lowest *T*‐scores. Over 1 year of therapy, the probabilities of achieving BMD levels above the osteoporosis range at total hip and lumbar spine were much higher with romosozumab than alendronate. When selecting initial treatment for patients with osteoporosis, especially in those with a high risk of imminent or long‐term fragility fracture, the BMD at baseline and probability of achieving their BMD goal should be considered.

## Disclosures

CD, ZW, ZY: Employees of Amgen. CL: Employee and stockholder of UCB Pharma. EH: Consulting/speaker fees from Amgen, UCB Pharma, Lilly, Ipsen, Theramex, AgNovos, and Alexion. EL: Consulting/speaker fees from Amgen, Lilly, Theramex, and UCB Pharma. FC: Received institutional grants and research support from Amgen; has served as a consultant for Amgen and Radius Health; and has served on the speakers' bureaus for Amgen and Radius Health. FB: Consulting/speaker fees from Amgen, UCB Pharma, Lilly, Abiogen, Astellas, Janssen, and Chiesi Farmaceutici. J‐EBJ: Advisory boards: Amgen, Eli Lilly, UCB Pharma, and Gedeon Richter; lectures: Amgen, Eli Lilly, UCB Pharma, Gilead, and Otsuka. PP: Consulting/speaker fees from Amgen, UCB Pharma, Lilly, Kyowa and Kirin. SF: Received research and/or consulting grants from Amgen, UCB Pharma, Radius, Agnovos, Alexion, and Gedeon Richter. SRC: Consultant and honoraria for speaking from Amgen.

## Data Sharing Statement

Underlying data from this manuscript may be requested by qualified researchers 6 months after product approval in the US and/or Europe, or global development is discontinued, and 18 months after trial completion. Investigators may request access to anonymized IPD and redacted study documents, which may include raw data sets, analysis‐ready data sets, study protocol, blank case report form, annotated case report form, statistical analysis plan, data set specifications, and clinical study report. Before use of the data, proposals need to be approved by an independent review panel at www.Vivli.org and a signed data sharing agreement will need to be executed. All documents are available in English only, for a prespecified time, typically 12 months, on a password‐protected portal.

### Peer Review

The peer review history for this article is available at https://publons.com/publon/10.1002/jbm4.10546.

## Supporting information


**Appendix S1**. Supplementary Information
