# Supplementary material for: Romosozumab Followed by Antiresorptive Treatment Increases the Probability of Achieving Bone Mineral Density Treatment Goals
Source: JBMR Plus. 2021 Oct 6;5(11):e10546. doi: 10.1002/jbm4.10546 (PMC8567484; doi:10.1002/jbm4.10546)
Supplement: Supplementary file 1 — Appendix S1. Supplementary Information [file JBM4-5-e10546-s001.docx]

# SUPPLEMENTARY INFORMATION

**Table S1.** Probabilities of achieving a T-score ≥−2.0 in the total hip and lumbar spine at Years 3 and 1 in patients with baseline T-scores equal to –2.7, –3.0, and –3.5

| Treatment | Baseline  T-score | Site of BMD Measurement at Year 3 | Probability of  T-score ≥−2.0  at 3 Years, % (95% CI) | Site of BMD Measurement at Year 1 | Probability of  T-score ≥−2.0  at 1 Year, % (95% CI) |
| --- | --- | --- | --- | --- | --- |
| ALN  (N=1825) | –2.7 | Total hip  (n=735) [a] | 2.3 (1.1, 4.4) | Total hip  (n=1778) [c] | 0.3 (0.1, 0.6) |
|  | –3.0 |  | 0.2 (0.1, 0.6) |  | 0.0 (0.0, 0.0) |
|  | –3.5 |  | 0.0 (0.0, 0.0) |  | 0.0 (0.0, 0.0) |
|  | –2.7 | Lumbar spine  (n=719) [b] | 39.4 (32.7, 46.6) | Lumbar spine  (n=1714) [d] | 7.8 (5.6, 10.9) |
|  | –3.0 |  | 14.6 (10.4, 20.2) |  | 1.4 (0.8, 2.4) |
|  | –3.5 |  | 1.8 (0.9, 3.6) |  | 0.1 (0.0, 0.2) |
| Romo/ALN  (N=1814) | –2.7 | Total hip  (n=748) [a] | 12.4 (9.2, 16.4) | Total hip  (n=1773) [c] | 4.7 (3.4, 6.5) |
|  | –3.0 |  | 3.0 (1.8, 5.0) |  | 0.6 (0.4, 1.1) |
|  | –3.5 |  | 0.2 (0.1, 0.6) |  | 0.0 (0.0, 0.1) |
|  | –2.7 | Lumbar spine  (n=730) [b] | 68.3 (62.4, 73.7) | Lumbar spine  (n=1715) [d] | 67.2 (62.8, 71.3) |
|  | –3.0 |  | 47.1 (41.5, 52.7) |  | 37.9 (33.9, 42.0) |
|  | –3.5 |  | 16.9 (12.9, 21.7) |  | 7.5 (5.7, 9.9) |
| Romo/Dmab  (N=3234) | –2.7 | Total hip  (n=2837) [a] | 27.9 (25.5, 30.5) | Total hip  (n=3186) [c] | 5.5 (4.5, 6.7) |
|  | –3.0 |  | 6.3 (5.1, 7.7) |  | 0.6 (0.4, 0.9) |
|  | –3.5 |  | 0.4 (0.2, 0.5) |  | 0.0 (0.0, 0.0) |
|  | –2.7 | Lumbar spine  (n=2791) [b] | 88.7 (86.6, 90.5) | Lumbar spine  (n=3140) [d] | 69.9 (66.7, 72.8) |
|  | –3.0 |  | 70.7 (67.9, 73.4) |  | 34.3 (31.3, 37.3) |
|  | –3.5 |  | 25.3 (22.4, 28.5) |  | 4.1 (3.2, 5.4) |

N = Number of patients with total hip BMD values at baseline and at least one evaluable total hip BMD value at month 12 or month 36, or with lumbar spine BMD values at baseline and at least one evaluable lumbar spine BMD value at month 12 or month 36. [a] Number of patients with evaluable total hip BMD value at baseline and month 36; [b] Number of patients with evaluable lumbar spine BMD value at baseline and month 36; [c] Number of patients with evaluable total hip BMD value at baseline and month 12; [d] Number of patients with evaluable lumbar spine BMD value at baseline and month 12. ALN: alendronate; BMD: bone mineral density; CI: confidence interval; Dmab: denosumab; Romo: romosozumab.
